# Preventive immunization of aged and juvenile non-human primates to beta-amyloid

**DOI:** 10.1186/1742-2094-9-84

**Published:** 2012-05-03

**Authors:** Julia Kofler, Brian Lopresti, Chris Janssen, Anita M Trichel, Eliezer Masliah, Olivera J Finn, Russell D Salter, Geoffrey H Murdoch, Chester A Mathis, Clayton A Wiley

**Affiliations:** 1 Department of Pathology, University of Pittsburgh, 200 Lothrop Street, Pittsburgh, PA 15213, USA; 2 Department of Radiology, University of Pittsburgh, 200 Lothrop Street, Pittsburgh, PA 15213, USA; 3 Department of Immunology, University of Pittsburgh, 200 Lothrop Street, Pittsburgh, PA 15213, USA; 4 Division of Laboratory Animal Resources, University of Pittsburgh, 200 Lothrop Street, Pittsburgh, PA 15213, USA; 5 Department of Neurosciences, University of California San Diego, 9500 Gilman Drive, La Jolla, CA 92093, USA

**Keywords:** Alzheimer’s disease, Beta-amyloid, Oligomers, Active immunization, Preventive immunotherapy, Neuroinflammation, Microglia, Classical and alternative activation, Immunosenescence

## Abstract

**Background:**

Immunization against beta-amyloid (Aβ) is a promising approach for the treatment of Alzheimer’s disease, but the optimal timing for the vaccination remains to be determined. Preventive immunization approaches may be more efficacious and associated with fewer side-effects; however, there is only limited information available from primate models about the effects of preclinical vaccination on brain amyloid composition and the neuroinflammatory milieu.

**Methods:**

Ten non-human primates (NHP) of advanced age (18–26 years) and eight 2-year-old juvenile NHPs were immunized at 0, 2, 6, 10 and 14 weeks with aggregated Aβ_42_ admixed with monophosphoryl lipid A as adjuvant, and monitored for up to 6 months. Anti-Aβ antibody levels and immune activation markers were assessed in plasma and cerebrospinal fluid samples before and at several time-points after immunization. Microglial activity was determined by [^11^C]PK11195 PET scans acquired before and after immunization, and by post-mortem immunohistochemical and real-time PCR evaluation. Aβ oligomer composition was assessed by immunoblot analysis in the frontal cortex of aged immunized and non-immunized control animals.

**Results:**

All juvenile animals developed a strong and sustained serum anti-Aβ IgG antibody response, whereas only 80 % of aged animals developed detectable antibodies. The immune response in aged monkeys was more delayed and significantly weaker, and was also more variable between animals. Pre- and post-immunization [^11^C]PK11195 PET scans showed no evidence of vaccine-related microglial activation. Post-mortem brain tissue analysis indicated a low overall amyloid burden, but revealed a significant shift in oligomer size with an increase in the dimer:pentamer ratio in aged immunized animals compared with non-immunized controls (*P* < 0.01). No differences were seen in microglial density or expression of classical and alternative microglial activation markers between immunized and control animals.

**Conclusions:**

Our results indicate that preventive Aβ immunization is a safe therapeutic approach lacking adverse CNS immune system activation or other serious side-effects in both aged and juvenile NHP cohorts. A significant shift in the composition of soluble oligomers towards smaller species might facilitate removal of toxic Aβ species from the brain.

## Background

Passive and active immunization against beta-amyloid (Aβ) has been proven efficacious in removing Aβ aggregates from the brain [1-3]. In rodents, this clearance was associated with cognitive improvement in many but not all studies [4-9], whereas only marginal differences were noted in cognitive outcome measures between vaccinated and non-vaccinated humans [10-12]. Moreover, serious side-effects including meningoencephalitis in a subset of patients and the development of vasogenic edema have complicated human immunization studies [13,14].

One possible explanation for the lack of success in human studies might be the timing of therapeutic intervention. Aβ species begin to accumulate in the brain many years to decades before cognitive symptoms develop [15]. At the time of enrollment into vaccination studies, patients with a clinical diagnosis of probable Alzheimer’s disease (AD) have already developed secondary neurodegenerative changes including tau accumulation, synaptic pathology, neu-ronal loss, angiopathy, and neuroinflammation, which may be irreversible and thus unaffected by reducing the brain Aβ burden [13,16]. Therefore, if immunization therapy were to be efficacious, it would probably need to be administered earlier. Studies in transgenic mouse models have already shown that Aβ accumulation in the brain and cognitive deficits can be markedly diminished by preventive immunization strategies initiated before the onset of significant Aβ deposition [1,2,8]. In addition, vaccination at a time when the brain amyloid burden is still low may reduce the risk of toxic side-effects mediated by soluble amyloid species that might possibly be released from disaggregated plaques, and may decrease the risk of cerebral amyloid angiopathy-associated microhemorrhages promoted by amyloid shift from parenchyma to the vasculature [17].

Based on these considerations, there are increased efforts to start immunization during a crucial time window of preclinical AD when Aβ pathology is still minimal [13,16,18]. The development of improved biomarker panels and ima-ging paradigms (Pittsburgh Compound B (PiB) PET scans [19]) may soon allow the more accurate identification of individuals at risk who may benefit most from early intervention. Biomarkers of interest include CSF Aβ_42_, phospho-tau and tau levels [20,21] and more novel markers such as YKL40, which may help distinguish between control, mild cognitive impairment (MCI) and mild AD [18,22,23].

To date, preventive immunization approaches have not been studied in a non-human primate (NHP) model, therefore we sought to evaluate the effects of immunization on the aging NHP brain before the onset of a significant Aβ burden by addressing the following questions:

1) Does active Aβ vaccination in an individual with low amyloid burden alter the level and composition of Aβ species in the brain or CSF?

2) Is preventive immunization associated with microglial activation or shifts in the neuroinflammatory milieu?

3) Does immunosenescence affect the efficacy of preventive Aβ immunization in an aging population?

## Methods

### Animals

All animals were housed and maintained according to strict standards of the Association for Assessment and Accreditation of Laboratory Animal Care, and experiments were approved by the University of Pittsburgh Institutional Animal Care and Use Committee.

In total, 10 aged macaques (aged 18 to 26 years; 5 rhesus, 1 cynomolgus and 4 pigtailed macaques; 2 males, 8 females) and 8 juvenile pigtailed macaques (1 to 2 years; all male) were included in the immunization protocol. Archival brain tissue and fluid samples from age-matched aged macaques (18–21 years; 4 rhesus, 2 cynomolgus; all male) and juvenile macaques (1–2 years; 11 pigtailed; 5 males, 6 females) were used as non-immunized control samples in some experiments as detailed below. Unless otherwise indicated, experiments were performed on all animals in each treatment cohort.

### Vaccine preparation

Beta-amyloid 1–42 (Aβ_42_; American Peptide Company, Sunnyvale, CA, USA) was dissolved in sterile PBS (1 mg/ml), mixed by vortex for 5 minutes and subsequently incubated at 37°C for 24 hours to allow for aggregation [2]. Synthetic monophosphoryl lipid A (MPL; InvivoGen, San Diego, CA, USA) was solubilized in DMSO and diluted in sterile water for a final concentration of 0.5 mg/ml. Single vaccine aliquots were prepared by mixing 100 μl of Aβ stock solution (equal to 100 μg) with 200 μl of MPL adjuvant solution, and these were then stored at −20°C until use.

To assess the animals’ general immune responsiveness, a control vaccine consisting of tetanus and diphtheria toxoid (Massachusetts Public Health Biologic Laboratories, Boston, MA, USA) was administered in accordance with the manufacturer’s dosing instructions.

### Study timeline

Amyloid vaccines were administered subcutaneously at 0, 2, 6, 10, and 14 weeks. Tetanus–diphtheria vaccines were given at 0 and 4 weeks in the contralateral appendage. EDTA plasma and serum samples were collected before the first vaccination and every 2 weeks thereafter throughout the study period. CSF samples were drawn before vaccination and either every 2 weeks (aged animals) or 4 weeks (juveniles) throughout the study period. All samples were collected in the morning, between 08.00 and 10.00 AM, to minimize circadian fluctuations. Blood and CSF samples were stored in aliquots at −80°C until further analysis.

Animals were monitored for at least 16 weeks. If no rise in anti-Aβ antibody titer had occurred by that time-point, the study period was extended for another 8 weeks for a total of 24 weeks. Any animal that did not develop a measurable antibody concentration by 24 weeks was classified as a non-responder.

[^11^C]PK11195 PET scans were performed at baseline and after development of an antibody response (around weeks 12 to 16). In non-responders, the follow-up scan was performed at the end of the study period (weeks 20 to 24). PET scans could not be performed on two of the aged animals for logistical reasons. One additional aged animal received a baseline scan but not a post-immunization scan because of technical difficulties.

### (R)-[N-methyl-^11^C]PK11195 ([^11^C]PK11195) positron emission tomography imaging

[^11^C]PK11195 was synthesized at the University of Pittsburgh PET Facility as previously described [24]. [^11^C]PK11195 PET scans were acquired with the use of a PET scanner (either microPET P4 or ECAT HR+; Siemens Medical Systems, Knoxville, TN, USA) operating in three-dimensional (volume imaging) mode. For smaller animals (<10 kg), the microPET P4 scanner was used for imaging. This scanner has a 220 mm animal port that simultaneously acquires 63 image planes over an 80 mm axial field of view (FOV) with a maximum reconstructed image spatial resolution of ~1.8 mm full width at half maximum (FWHM) [25]. Animals too large to be accommodated by the microPET P4 system were imaged using the ECAT HR + scanner, which is a clinical PET tomograph that simultaneously acquires 63 image planes over a 152-mm axial FOV with an intrinsic spatial resolution of ~4.5 mm FWHM.

Before radiotracer injection, transmission scans were performed for attenuation correction of PET emission data. Transmission scans were conducted using either a ~130 MBq (3.5 mCi) rotating ^57^Co point source (microPET P4) or ^68^Ga^68^Ge rod sources. Attenuation correction factors were calculated from back-projection and segmentation of the transmission image (microPET P4) or measured directly (ECAT HR+). Following the transmission scan, 311 ± 52 MBq (8.4 ± 1.4 mCi) (range 255 to 429 MBq; 6.9 to 11.6 mCi) of [^11^C]PK11195 with high specific activity (>55.5 GBq/μmol (1.5 Ci/μmol) at time of injection(TOI)) was injected intravenously into each macaque over a period of 30 seconds. PET data were acquired for 90 minutes in a dynamic series of 33 frames and corrected for attenuation, dead time, scatter, and radioactive decay. [^11^C]PK11195 PET scans of each macaque were co-registered to the baseline PET scan using the normalized mutual information algorithm implemented in the PMOD software package (PMOD Technologies, Zurich, Switzerland). Volumes of interest (VOI) were defined bilaterally on summed PET images (0 to 8 minutes post-injection) for cerebral cortex (combined region covering the frontal, lateral temporal, and parietal cortices), thalamus, cerebellum, and subcortical white matter. VOIs were applied to the dynamic emission data to generate regional time–activity concentration curves. Regional radioactivity concentrations were summed over the post-injection interval of 10 to 60 minutes, and normalized to that in the subcortical white matter (SWM) to estimate the specific retention of [^11^C]PK11195 as previously described [26,27].

### [^11^C]Pittsburgh compound- B (^11^C]PiB) positron emission tomography imaging

[^11^C]PiB was synthesized at the University of Pittsburgh PET Facility using the captive solvent method as previously described [28]. [^11^C]PiB-PET images were acquired on both microPET P4 and ECAT HR + PET scanners as described above for the [^11^C]PK11195 studies. Following the completion of the transmission scan, 377 ± 85 MBq (10.2 ± 2.3 mCi) (range: 296 to 537 MBq; 8.0 to 14.5 mCi]) of high specific activity (>22.2 GBq/μmol (0.6 Ci/μmol) at TOI) [^11^C]PiB was injected intravenously over 30 seconds. PET data were acquired for 90 minutes in a dynamic series of 33 frames, and corrected for attenuation, dead time, scatter, and radioactive decay. Regional [^11^C]PiB retention measures for frontal cortex, temporoparietal cortex, thalamus, and pons were determined using the late scan (40 to 90 minutes) standardized uptake value ratio (SUVR) method and the cerebellum as reference as previously described [29].

### Anti-Aβ_40_, anti-Aβ_42_ and anti-toxoid IgG antibody measurements

Anti-Aβ antibody levels in plasma and CSF were measured by ELISA using published protocols with minor modifications [30-32]. In brief, 96-well ELISA plates (Immulon 2HB; ThermoFisher Scientific, Pittsburgh, PA, USA) were coated overnight with solubilized Aβ_40_ or Aβ_42_ (American Peptide Company, Sunnyvale, CA, USA) in bicarbonate coating buffer, pH 9.6 (Bethyl, Montgomery, TX, USA). After washing twice with washing solution (0.1% Tween-20 in PBS), the wells were blocked first with blocking buffer (SuperBlock; ThermoFisher Scientific) followed by an additional blocking step with 5% milk in PBS-Tween. Samples (100 μl per well) were then incubated for 2 hours at room temperature. After three washes, horseradish peroxidase-conjugated anti-monkey IgG detection antibody (1:30,000; Rockland, Gilbertsville, PA, USA) was added to each well for 1 hour. After five washes, 100 μl of tetramethylbenzidine substrate (Sigma, St. Louis, MO, USA) was dispensed into each well. After 15 minutes, the developing color reaction was stopped with 50 μl of 1 mol/l HCl solution. Optical density was immediately measured at 450 nm using a microplate reader (ELx800; BioTek, Winooski, VT, USA).

Serum anti-tetanus toxoid antibody levels were measured by ELISA (IBL, Minneapolis, MN, USA). Similar results were achieved using the anti-human detection antibody supplied with the kit as with an anti-monkey IgG detection antibody, thus all subsequent experiments were performed using the reagents in the kit. Results are expressed as IU/ml.

### Aβ_40_, Aβ_42_, total tau, and YKL40 measurements

Aβ_40_, Aβ_42_, and YKL40 concentrations were measured in CSF and plasma using ELISA kits according to the manufacturers’ protocols (Aβ1-40 and Aβ1-42 (Invitrogen, Camarillo, CA, USA) and YKL40 (Quidel Corporation, San Diego, CA, USA)). CSF samples were also assessed for total tau concentration by ELISA (Invitrogen). In addition to absolute concentrations, the tau to Aβ_42_ ratio was calculated for CSF samples.

### Multiplex analysis of neuroimmune marker

Commercial cytokine assay kits (Procarta; Panomics, Fremont, CA, USA) were used to simultaneously detect one human-specific cytokine (macrophage colony-stimulating factor; M-CSF) and 30 NHP-specific proteins in CSF samples. These assays were based on detection technology (xMAP; Luminex Corp., Austin, TX, USA) that uses beads to quantitatively measure multiple cytokines in a small amount of sample. Cytokines were quantified at baseline before vaccination and weeks 4 and 16 after immunization. CSF samples were diluted 1:2 in buffer (Non-human Primate Bodily Fluid Buffer; Panomics) and processed according to the manufacturer’s recommendations. The plate was read at the University of Pittsburgh Cancer Institute Luminex Facility (Bio-Plex reader Luminex 100, Luminex Corp.). Analyte concentrations were calculated based on the respective standard curve for each analyte.

### Determination of Aβ levels in the brain by immunoblot and ELISA

Tissue fractionation and extraction was performed as previously described [33]. Briefly, the frontal cortex (0.1 g) was homogenized in 0.4 ml of buffer containing NaCl–P_i_ pH 7.4, 0.32 mol/l sucrose, 50 mmol/l Hepes, 25 mmol/l MgCl2, 0.5 mmol/l dithiothreitol, and phosphatase and protease inhibitor cocktails (Calbiochem, San Diego, CA, USA). The samples were separated by centrifugation for 10 minutes at 1000 *g* and 4°C. Supernatants were transferred into appropriate ultracentrifuge tubes, and the pellets were rehomogenized in 0.3 mL of buffer, then separated again by centrifugation for 10 minutes at 1000 *g* and 4°C. The second supernatant was collected and combined with the first supernatant and separated by centrifugation for 1 hour at 100,000 *g* and 4°C. This final supernatant was collected to serve as the cytosolic fraction, and the remaining pellet was resuspended in 0.2 mL of buffer and rehomogenized to give the membrane fraction. The bicinchoninic acid protein assay was used to determine the protein concentration of the samples.

For immunoblot analysis, samples (20 μg) were loaded onto a 4 to 12% Bis-Tris gel (Invitrogen, Carlsbad, CA, USA) and run with MES–SDS running buffer (NuPAGE; Invitrogen) at 200 V for 45 minutes on ice. Gels were transferred to a 0.2 μm nitrocellulose membrane, in transfer buffer (1 l Tris-glycine buffer containing 20% methanol) at 400 mA for 1.5 hours. Membranes were boiled in phosphate-buffered saline for 5 minutes, blocked with 3% BSA in Tris-buffered saline and 0.05% Tween 20 (TBS-T) for 1 hour, and incubated overnight at 4°C with anti-Aβ antibody (6E10, Signet Laboratories, Dedham, MA, USA). Membranes were washed in TBS-T for 1 hour, and incubated in secondary antibody for 1 hour at room temperature. Membranes were washed for 30 minutes in TBS-T and then for five minutes in TBS alone. Detection was carried out with a commercial reagent (Western Lightning Chemiluminescence Reagent Plus; PerkinElmer, Waltham, MA, USA) and visualized by enhanced chemiluminescence. Membranes were analyzed with a Versadoc XL imaging apparatus (BioRad, Hercules, CA, USA). Analysis of actin levels (C4, Millipore, Temecular, CA, USA) was used as a loading control.

Aβ_42_ concentrations were also measured by ELISA in accordance with the manufacturer’s protocol (Invitrogen, Camarillo, CA, USA).

### Histopathologic and immunohistochemical evaluation

Animals were killed and perfused with saline, the brains were then removed immediately and processed for neuropathologic analysis as described previously [34]. Briefly, brains were bisected sagittally, then the left half was fixed in 10% formalin and tissue blocks were embedded in paraffin wax, while the right half was micro-dissected and snap-frozen at −80°C. Sections of brain were stained with hematoxylin and eosin, or immunostained for the macrophage–microglia-associated protein Iba-1 (1:500, Wako Chemicals USA, Richmond, VA, USA) and the T-cell marker CD3 (polyclonal, 1:500, Dako, Carpenteria, CA, USA). The presence of amyloid pathology was evaluated by Bielschowsky silver stain and immunohistochemical stains for Aβ (clone 6F/3D, 1:100; Dako (formic acid pre-treatment)) and total tau (polyclonal antibody, 1:200; Dako). To assess if vaccination-induced anti-Aβ antibodies bind to Aβ plaques, colabeling for Aβ and anti-monkey IgG (1:500, Rockland, Gilbertsville, PA) was performed.

Prussian blue iron stain was used to screen for microhemorrhages. Slides were dewaxed, rehydrated, and immersed in 10% potassium ferrocyanide for 5 minutes, followed by immersion in equal parts of 20% hydrochloric acid and 10% potassium ferrocyanide for 30 minutes. Slides were counterstained with Nuclear fast red.

### Quantitative image analysis

Amyloid plaque burden, vascular amyloid, and neurofibrillary tangle density were classified using a semi-quantitative scale (none, minimal, mild, moderate, severe). Diffuse and neuritic plaque density was scored as none, minimal, mild, moderate, or severe. Following CERAD (Consortium to Establish a Registry for Alzheimer's Disease) guidelines, plaque density was assessed in the most severely affected area of each evaluated brain region [35].

Microglial cell density was assessed by single-label Iba-1 immunohistochemistry without counterstaining. Images (×200 magnification) were taken of random fields in the mid-frontal cortex (n = 10 fields), hippocampus (5), entorhinal cortex (5), corpus callosum (3), subcortical mid-frontal white matter (3), and periventricular white matter (3). The area fraction immunopositive for Iba-1 was measured using Image J software (http://rsb.info.nih.gov/ij/) for each image, and the mean value calculated for each area. The three white-matter regions were combined into one area.

### Quantitative real-time polymerase chain reaction

RNA was isolated from frozen frontal cortex samples using a commercial kit (RNeasy Mini Kit; Qiagen, Valencia, CA, USA) in accordance with the manufacturer’s instructions. RNA concentrations were measured by spectrophotometry (Nanodrop 1000; ThermoScientific, Wilmington, DE, USA). After conversion of RNA into cDNA (RETROscript protocol; Ambion, Austin, TX, USA), quantitative real-time PCR was performed (QuantiTect SYBR Green PCR kit; Qiagen) and NHP-specific primers for the microglial markers CD68 and Iba-1, the astrocyte marker glial fibrillary acidic protein (GFAP), the classical macrophage activation (M1) markers CXCL9, CXCL10, prostaglandin-endoperoxide synthase (PTGS)2 (cyclooxygenase 2), indoleamine 2,3 dioxygenase (IDO) and CCR7, and the alternative macrophage activation markers mannose receptor 1 (MRC1:CD206) and CCL17 (Table 1). Primers were designed using Primer3 software (http://primer3.sourceforge.net/). Samples were run on a real-time PCR system (StepOnePlus; Applied Biosystems, Foster City, CA, USA) using standard cycling conditions. Results are presented as relative gene expression normalized to glyceraldehyde 3-phosphate dehydrogenase (GAPDH) as internal control (2^-dCT^ where dCt = Ct gene of interest − Ct internal control).

**Table 1:** Primer sequences for microglia and astrocyte cell markers and M1 or M2 activation markers

**Gene**	**NCBI reference sequence**	**Forward primer**	**Reverse primer**
*Iba-1*	NM_001047118.1	ccagggatttacagggagga	atcgccgtttccattaaggt
*CD68*	XM_001110126	cagcacagtggacattctcg	tgatgagaggcagcaagatg
*GFAP*	XM_001102095.2	aagctccaggatgaaaccaa	aacctcctcctcgtggatct
*CXCL9*	NM_001032936.1	taatgaggaagggtcgctgt	tttggctgacctgtttttcc
*CXCL10*	NM_001032892.1	ttgctgccttgtctttctga	tgatggccttagattctgga
*IDO*	NM_001077483.1	ccgtcaagtgtttcagcaaa	caggacgtcaaagcactgaa
*CCR7*	NM_001032884	gtggtggctctccttgtcat	gtaggcccacgaaacaaatg
*PTGS2*	XM_001107538.2	cccttgggtgtgaaaggtaa	gccctcgcttatgatctgtc
*MRC1*	NM_001193925	aaggaaaccatggacaatgc	ccatccatccaagcaaactt
*CCL17*	NM_001032852.1	cttctctgcagcacatccat	aacagatggccttgttctgg
*GAPDH*	NM_001195426	tggaaggactcatgaccaca	ttcagctcagggatgacctt

### Statistical analysis

Statistical analysis was performed using GraphPad Prism software (LaJolla, CA, USA). Comparisons between groups were performed using the Student’s *t*-test or, when data were not normally distributed, the non-parametric Mann–Whitney U-test. For data with more than one time-point, repeated measures two-way ANOVA was performed. *P* < 0.05 was considered significant.

## Results

### Antibody response to vaccinations

All eight juvenile animals completed the five-cycle vaccination protocol and at least 16 weeks of observation. Of the ten aged macaques, one was diagnosed with a colon carcinoma during the study period and was euthanized at week 6, after receiving three of five vaccine cycles.

**Figure 1:**
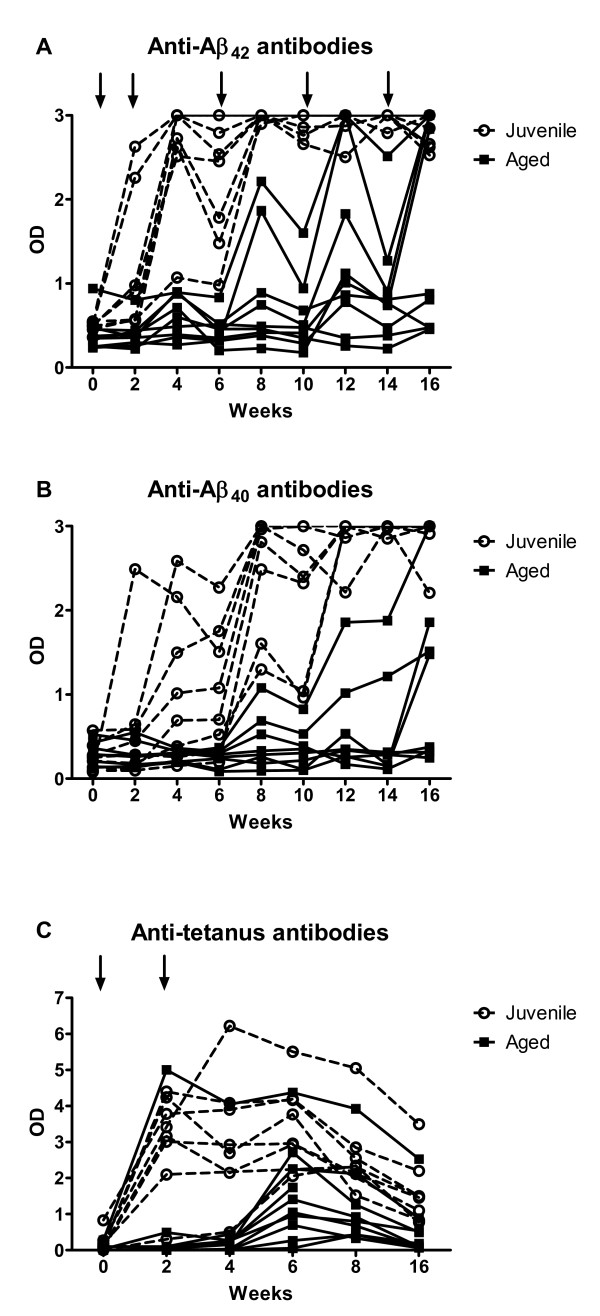
**Antibody response curves for plasma anti-Aβ**_**42**_**, anti-Aβ**_**40 **_**and anti-tetanus toxoid in individual juvenile (n = 8) and aged (n = 10) non-human primates.** Significant age-dependent differences in the immune response against **(A)** anti-Aβ_42_, **(B)** anti-Aβ_40 _and **(C)** tetanus toxoid were evident. Arrows indicate the time-points of (A) Aβ and (C) tetanus vaccine administrations.

All juvenile animals developed a strong antibody response against both Aβ_42_ and Aβ_40_ after the first or second vaccine dose, whereas only 80% of the ten aged animals developed detectable anti-Aβ_42_ antibodies (Figure 1A). In five of the eight aged responders, peak antibody concentrations approached levels similar to those in the juvenile cohort. The other three aged responders developed only low antibody levels. In all aged responders, antibody development was delayed compared with the juveniles, with minimal antibody response after the second vaccine dose and increasing titers after the third dose. Only 60 % of the aged animals developed anti-Aβ_40_ antibodies (Figure 1B). Repeated measures two-way ANOVA analysis showed a strong effect of age on the immune response for both anti-Aβ_42_ (*F* = 81.11; *P* < 0.0001) and anti-Aβ_40_ antibody levels (*F* = 70.04; *P* < 0.0001) with significant differences between age groups from week 4 until the end of the observation period. These results indicate significant age-dependent differences in the immune response to Aβ vaccination.

Age differences were also seen in the response to tetanus vaccination. Although all aged animals developed detectable antibody levels, the response was not as strong as in the juveniles, and required two vaccine-dosing cycles in all but one aged animal, whereas all juveniles produced high antibody titers already after the first dose (Figure 1 C). Repeated measures two-way ANOVA analysis showed a strong effect of age on the anti-tetanus antibody levels (*F* = 10.65; *P* < 0.0001) with significant differences between age groups from week 2 until week 6.

**Figure 2:**
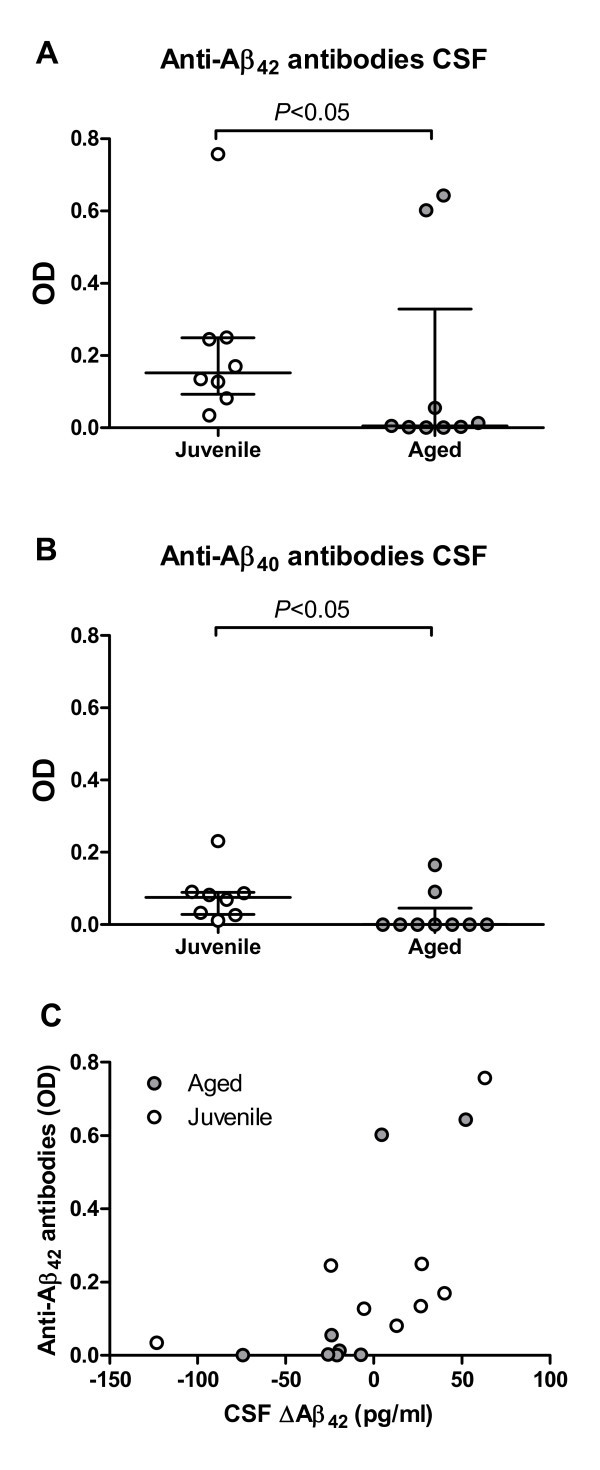
**Antibody levels in cerebrospinal fluid (CSF). (A)** Anti-Aβ_42_ and **(B)** anti-Aβ_40_ antibody levels were significantly lower at week 16 post-immunization in aged (n = 9) than in juvenile non-human primates (n = 8). Bars represent median and interquartile range. **(C)** Scatterplot displaying the difference (ΔAβ_42_) between CSF Aβ_42_ levels at week 16 and baseline . A significant positive correlation was seen for the aged (*P* < 0.05) but not the juvenile cohort.

Anti-Aβ antibody levels in the CSF were determined at week 16, when serum antibody concentrations were at peak levels (Figure 2). All juvenile animals contained detectable anti-Aβ_40_ and Aβ_42_ antibodies in the CSF, although the levels were much lower than in the serum. By contrast, anti-Aβ_42_ antibodies were seen in only 44% of the aged animals, and anti-Aβ_40_ antibodies in 22%. A CSF samples was unavailable for one aged animal at week 16.

IgG immunoglobulins were purified from sera of animals with a positive anti-Aβ immune response using protein A spin columns (ThermoFisher, Pittsburgh, PA, USA) and were found to stain amyloid plaques of a human brain from a patient with AD, confirming the specificity of the vaccination-induced antibodies (data not shown).

### Aβ_40_, Aβ_42_ and tau levels in cerebrospinal fluid and plasma

To determine the normal range of Aβ_40_, Aβ_42_ and tau in the CSF of macaques, we first analyzed pre-immunization samples from all aged and juvenile macaques and also additional CSF samples from aged and juvenile control animals. No differences were seen between young and old animals for Aβ_40_ and Aβ_42_ levels (Table 2). The results of the tau assay revealed a non-significant trend towards higher levels in the cohort of aged macaques (*P* = 0.06).

**Table 2:** **Baseline Aβ**_**40**_**, Aβ**_**42 **_**and total tau levels in cerebrospinal fluid and plasma of juvenile and aged NHPs (median and interquartile range)**

	**Juvenile animals (n = 19)**	**Aged animals (n = 16)**	***P *-value**
Cerebrospinal fluid			
Aβ_40_, pg/ml	1494 (923 to 2077)	1514 (971 to 2145)	NS
Aβ_42_, pg/ml	78.4 (65.3 to 118.4)	107.4 (59.8 to 162.1)	NS
Tau, pg/ml	131.4 (89.4 to 168.2)	249.0 (94.2 to 345.2)	0.06
Tau/Aβ_42_	1.48 (0.84 to 1.94)	1.84 (1.20 to 3.40)	NS
Plasma			
Aβ_40_, pg/ml	16.0 (15.1 to 21.0)	15.1 (11.4 to 18.8)	NS
Aβ_42_, pg/ml	ND	ND	

ND, not detected; NS, not significant.

**Figure 3:**
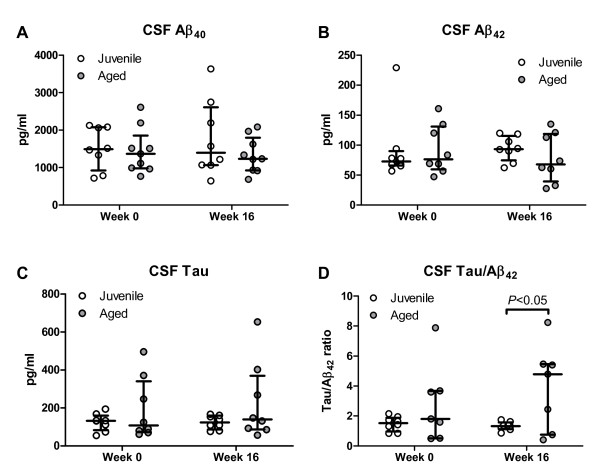
**Concentrations in cerebrospinal fluid of amyloid and tau proteins (A–C)** Concentrations of (A) Aβ_40_, (B) Aβ_42_ and (C) total tau before (week 0) and after (week 16) immunization. Bars represent median and interquartile range. No significant differences were seen between aged (n = 9) and juvenile animals (n = 8) or between baseline and post-immunization levels for either cohort. **(D)** However, a composite score, the tau:Aβ_42_ ratio showed a significant difference between juvenile and aged animals after, but not before, vaccination.

After immunization, average Aβ_40_, Aβ_42_ and tau CSF levels were not significantly different from baseline levels for either aged or juvenile animals (Figure 3), but there was marked variability in individual animals between pre- and post-immunization levels. A small overall decrease in CSF Aβ_42_ and increase in tau concentrations in the aged cohort resulted in a significant difference in the post-immunization tau/Aβ_42_ ratio compared with juveniles (*P* < 0.05).

Next, we wanted to determine if changes in CSF Aβ_42_ concentrations (difference between levels at week 16 post-immunization and baseline; ΔAβ_42_) were linked to the immune response. This analysis showed a positive correlation between ΔAβ_42_ and week 16 CSF anti-Aβ_42_ antibody levels, which was significant for the entire cohort of immunized animals (*P* < 0.05) and the subgroup of aged animals (*P* < 0.05) but not for the subgroup of juvenile animals. A stronger CSF humoral immune response was associated with increasing Aβ_42_ levels in the CSF, whereas declining Aβ_42_ levels were seen more frequently in animals with low or undetectable CSF anti-Aβ_42_ antibodies. No correlation was seen between ΔAβ_42_ and peak plasma anti-Aβ_42_ antibody levels for either aged or juvenile animals.

Pre-immunization plasma concentrations of Aβ_40_ were similar in juvenile and aged animals (Table 2). No significant changes were seen after immunization in either age group. Plasma Aβ_42_ levels were below the detection limit of our assay before and after immunization in both juvenile and older macaques.

### Aβ content in the brain

#### Histopathologic evaluation

Post-mortem histopathologic analysis was performed on all ten immunized aged animals and on six aged non-immunized control animals. Five immunized and three non-immunized animals exhibited mild focal Aβ deposits in their brains, mostly in the form of diffuse plaques, but rare neuritic plaques were also seen (Table 3). Only one of the animals had a focus of moderately dense diffuse plaques. None of the brains exhibited widespread Aβ deposits. In six animals, the Aβ pathology was restricted to the cortex, another animal had amyloid deposits in both cortex and caudate, and one other had Aβ plaques present exclusively in the head of the caudate nucleus. Co-labeling for Aβ and IgG did not identify any antibodies bound to Aβ plaques. In two brains, very focal vascular amyloid deposits were noted, which in one case involved very focally leptomeningeal and nearby cortical vessels, and in the second case, affected a very rare parenchymal small vessel. Only one of the non-immunized animals exhibited tau pathology in the form of rare neurofibrillary tangles in the subiculum, CA1 sector of the hippocampus and frontal cortex. No apparent differences in the degree of amyloid pathology were noted between the three macaque strains.

**Table 3:** Post-mortem ELISA Aβ42 levels and histopathologic evaluation of amyloid and tau pathology of immunized and non-immunized aged non-human primates

**Animal ID (age, years) (species, sex)**	**Immune response**	**Aβ**_**42**_**content frontal Cx, pg/ml**	**Diffuse plaques**	**Neuritic plaques**	**Vascular amyloid**	**Neuro fibrillary tangles**	**Other pathologic findings**
**Immunized animals**							
CW06-423 (22) (R, M)	H	BD	0	0	0	0	Colon carcinoma
CW07-220 (22) (R, F)	H	BD	0/+ (frontal and insular cortex)	0/+ (insular cortex)	+	0	Mild liver steatosis
CW07-221 (21) (R, F)	H	BD	0	0	0	0	Multilocular biliary liver cysts; mild lung emphysema
CW07-222 (21) (R, F)	H	BD	0/+ (frontal cortex); + (caudate head)	0/+ (caudate head)	0	0	Non-necrotizing granulomatous lung disease
CW08-237 (20) (P, F)	H	BD	0	0	0	0	0
CW06-422 (20) (R, M)	L	BD	0	0	0	0	ND
CW08-236 (20) (P, F)	L	BD	0	0	0	0	Renal cortical cysts
CW08-238 (18) (P, F)	L	22.17	++ (patchy frontal cortex); 0/+ (CA1, entorhinal, temporal, insular cortex)	++ (frontal cortex);	0	0	Papillary renal cell carcinoma; focal atypical adenomatous hyperplasia of the lung
CW07-223 (26) (C, F)	N	BD	0/+ (frontal cortex)	0	0	0	ND
CW08-239 (20) (P, F)	N	BD	+ (caudate head)	+ (caudate head)	0	0	0
**Non-immunized animals**							
CW06-420 (18) (C, M)	N/A	BD	0/+ (frontal cortex)	0	0	++ (subiculum); + (CA1, frontal cortex)	ND
CW06-421 (18) (C, M)	N/A	24.61	+ (frontal and temporal cortex)	0	0/+	0	Renal oncocytoma; renal cortical cysts; needle tract injury of the spinal cord
CW08-232 (18) (R, M)	N/A	BD	0	0	0	0	ND
CW08-233 (19) (R, M)	N/A	BD	0	0	0	0	Organizing eosinophilic pneumonia
CW08-234 (21) (R, M)	N/A	BD	0/+ (frontal cortex)	0	0	0	ND
CW08-235 (19) (R, M)	N/A	BD	0	0	0	0	ND

0, absent; 0/+, minimal; +, mild; ++, moderate (reflecting most severely affected area in each brain region); BD, below detection limit; C, cynomolgus macaque; Cx, cortex; F, female; H, high responder; L, low responder; M, male; N, non-responder; N/A, not applicable; ND, not done; P, pigtailed macaque; R, rhesus macaque.

On neuropathologic examination, no additional brain lesions were seen, other than the presence of axonal spheroids and hemosiderin deposits in the globus pallidus of all animals, and variable degrees of vascular mineralization in the globus pallidus of four animals. No evidence of meningoencephalitis was seen in any of the immunized animals.

Immunohistochemical stains for CD3 highlighted rare perivascular and leptomeningeal T-cells in both immunized and non-immunized animals. In addition, focally increased numbers of intraparenchymal lymphocytes were noted in the white but not gray matter of some brains. There was no association between treatment group and the incidence and density of these parenchymal T-cells. Prussian blue iron stain did not reveal any microhemorrhages in the cortex of the aged immunized animals or the non-vaccinated control animals. Post-mortem pathologic examination confirmed the presence of a moderately differentiated mucinous adenocarcinoma in the right colon of the one animal that had to be killed before the end of the study period. In addition, significant age-related non-CNS findings were seen in several other animals (Table 3).

#### [^11^C]PiB positron emission tomography imaging

[^11^C]PiB scanning was performed in two non-immunized and six immunized aged macaques at baseline. No significant [^11^C]PiB-PET signal was detected in any of the analyzed brain regions, indicating a relatively low level of fibrillar Aβ in these brains [36].

#### ELISA and western blot analyses of amyloid load and oligomer species

Aβ load in the frontal cortex was also determined by ELISA and showed good correlation with the histopathologic results (Table 3). Only the two animals (one immunized, one non-immunized) with the highest number of plaques in the frontal cortex as determined by immunohistochemistry had Aβ_42_ levels above the detection limit of the ELISA. Even though rare diffuse plaques were seen focally in the frontal cortex of five other animals, their Aβ_42_ load remained below the detection limit of the ELISA.

**Figure 4:**
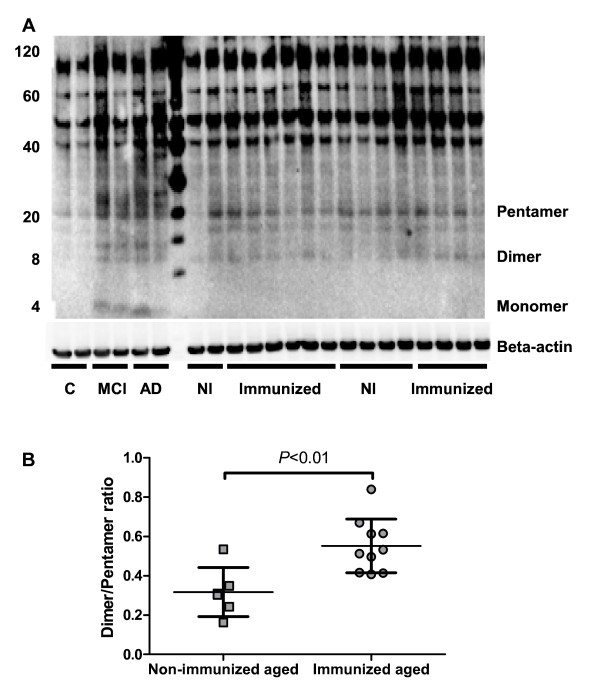
**Aβ composition in primate brains. (A)** Western blot analysis of Aβ oligomers in the membrane fraction of brain extracts from immunized (n = 10) and non-immunized aged non-human-primates (n = 6) using 6E10 antibody. The left lanes show human brain tissue samples without Alzheimer’s disease (AD), with mild cognitive impairment, and with AD for comparison. **(B**) Densitometric analysis showed a significant difference in the dimer to pentamer ratio in immunized compared with non-immunized aged animals. Bars represent mean ± SD.

The amyloid burden was very low and variable within each group, and there were no differences in overall amyloid load between immunized and non-immunized animals. However, further characterization of Aβ species in the frontal cortex by western blotting identified a significant shift in oligomer size in the membrane fraction with an increase in the dimer:pentamer ratio in immunized compared with non-immunized animals (*P* < 0.01; Figure 4). No correlation was found between strength of immune response as determined by peak anti-Aβ_42_ antibody levels and dimer:pentamer ratio. No clear oligomeric bands were seen on blots of the cytosolic fraction (data not shown).

### Assessment of microglial activation

#### [^11^C]PK11195 positron emission tomography imaging

**Figure 5:**
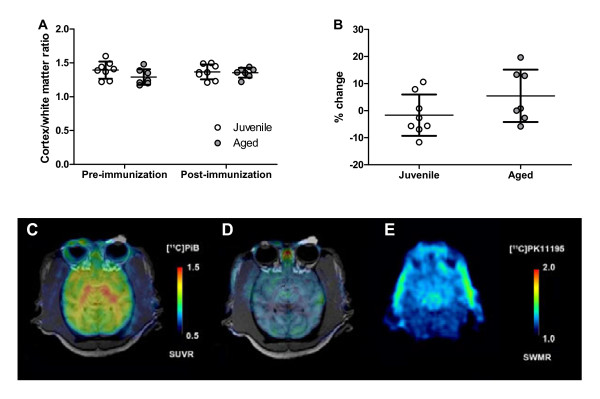
**Positron emission tomography imaging analysis. (A)** [^11^C]PK11195 cortex to subcortical white-matter ratio indicated no differences in microglial activation between juvenile (n = 8) and aged (n = 7) animals before or after immunization. **(B)** Comparison between pre- and post-immunization scans showed a trend towards increased [^11^C]PK11195 retention over time in aged animals. **(C**) Representative transaxial parametric image of [^11^C]PiB standardized uptake value ratio (SUVR) of an aged animal showed no evidence of significant amyloid deposition. The PET image is superimposed over a corresponding MRI image to aid anatomic orientation. **(D, E)**, Representative transaxial parametric images of [^11^C]PK11195 cortex to subcortical white-matter ratio (SWMR) revealed no significant microglial activation in (D) aged or (E) juvenile animals.

To investigate the microglial response to Aβ immunization *in vivo*, all juvenile and eight of the ten aged immunized animals underwent PET imaging using [^11^C]PK11195, a selective radioligand of the peripheral benzodiazepine receptor, also called the 18 kDa translocator protein (TSPO) [37]. Compared with pre-immunization studies, no significant changes were seen in any brain region between pre- and post-immunization scans, although there was a slight trend towards increased [^11^C]PK11195 retention over time in the aged but not the juvenile animals (Figure 5). Subgroup analysis of the aged animals did not identify any clear trends distinguishing immune responders from non-responders. As determination of regional [^11^C]PK11195 retention required a normalization step to white matter in this analysis, we were unable to assess whether there was a global increase in microglial activity in aged compared with juvenile animals. No association was seen between age and cortical [^11^C]PK11195 retention (relative to white matter), in contrast to recent reports of significant age-associated increases in [^11^C]PK11195 binding found in cognitively normal elderly humans [38].

### Microglial quantification by immunohistochemistry and real-time PCR

**Figure 6:**
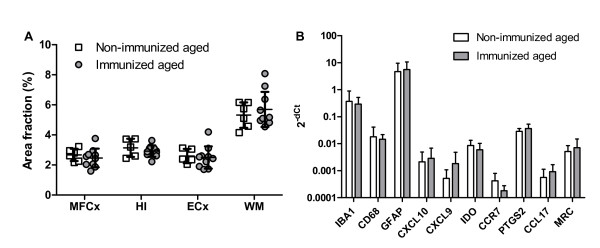
**Immunohistochemical and real-time (RT) PCR analyses. (A) **Immunohistochemical assessment of microglial density in mid-frontal cortex (MFCx), hippocampus (HI), entorhinal cortex (ECx) and white matter (WM), expressed as the Iba-1-positive area fraction. No differences were seen in any brain area between immunized (*n* = 10) and non-immunized aged non-human primates (NHPs) (*n* = 6). Bars represent mean ± SD. **(B)** Quantitative RT-PCR analysis of expression of microglial (CD68 and Iba-1) and astrocytic (GFAP) markers as well as indicators of classical (CXCL10, CXCL9, indoleamine 2,3 dioxygenase (IDO), CCR7, prostaglandin-endoperoxide synthase 2 (PTGS2)) and alternative (CCL17, mannose receptor 1 (MRC1) microglia activation. No significant differences were seen between immunized and non-immunized NHPs for any marker. Mean ± SD.

To further evaluate the effects of anti-Aβ immunization on microglial activity, we quantified the number of microglial cells by Iba-1 immunohistochemistry in several brain regions, including the mid-frontal cortex, entorhinal cortex, hippocampus, corpus callosum, and white matter. Because the juvenile immunized animals were not euthanized at the end of the study period, we were not able to further analyze this cohort of our animals. The brains from immunized aged animals were compared with a cohort of non-immunized age-matched controls. The density of microglial cells was significantly higher in white matter compared with all three gray matter regions analyzed (*P* < 0.05, respectively; Figure 6A), very similar to the human brain [39]. No differences were seen between immunized and non-immunized animals, or between immune responders and non-responders for any brain region. Animals with detectable Aβ by immunohistochemistry or ELISA had cortical microglial densities similar to those of animals without detectable plaques. These immunohistochemical findings are supported by the results of quantitative real-time PCR studies, which showed no differences in the levels of Iba-1, CD68 or the astrocytic marker GFAP (Figure 6B).

Taken together, the PET, immunohistochemistry and PCR data indicate that vaccination against Aβ was not associated with significant microglial activation in our cohort of animals. To further define microglial activation states and evaluate possible shifts in microglial phenotype, we performed quantitative real-time PCR studies for several markers of classical (M1) and alternative (M2) macrophage activation. As there are significant differences between rodents and humans in M1/M2 gene signatures [40], we first verified that our set of human M1/M2 markers is appropriately differentially regulated in NHP macrophages (unpublished data) before applying these markers to the brain samples of the current study. These experiments showed that markers of both M1 and M2 polarization are expressed in the brain (Figure 6B). No difference was seen between immunized and non-immunized aged animals, indicating that vaccination did not induce a shift in microglial activation state. No obvious differences were noted in microglial density or activation markers between different macaque strains, but group sizes were too small for definitive conclusions.

### Immune activation markers in the CSF

**Figure 7:**
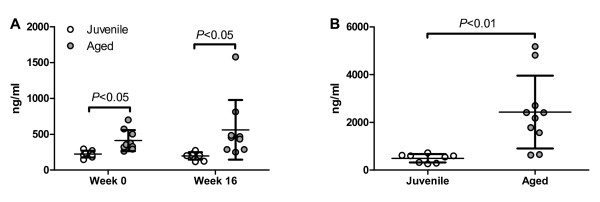
**Analysis of YKL40 release** There were significantly higher concentrations of YKL40 in (A) cerebrospinal fluid (CSF) and (B) plasma in aged (n = 10) than in juvenile (*n* = 8) animals at baseline in both fluid compartments. No significant changes were seen in the CSF after immunization (Aged, *n* = 9; juvenile, *n* = 8). Mean ± SD.

We have previously shown that YKL40 (chitinase 3-like protein 1; CHI3L1), a member of the glycosyl hydrolase family 18 lacking hydrolytic activity, is elevated in the CSF in a variety of acute and chronic neurologic diseases, and is most abundantly associated with astrocytes in regions of inflammatory cells [22]. To test if Aβ vaccination is also associated with significant glial activation, we measured YKL40 levels in the CSF before and at multiple time-points after vaccination. At all time-points, YKL40 concentrations were significantly higher in aged than in juvenile animals, but there was no significant change in YKL40 levels after immunization in either age group (Figure 7A). There was greater variability in post-immunization CSF YKL40 levels in aged animals, but no association between YKL40 levels and immune response was detected. Similar to the CSF results, plasma YKL40 levels were significantly higher in aged than in juvenile animals (Figure 7B). Two aged animals had substantially higher plasma YKL40 levels than the remaining cohort. Both of these animals were eventually diagnosed with malignancies: one with colon carcinoma, the other post-mortem with renal cell carcinoma.

**Figure 8:**
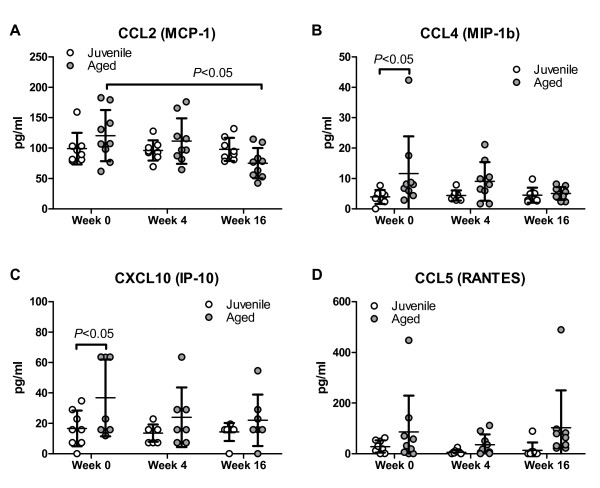
**Multiplex analysis of chemokines.** Cerebrospinal fluid (CSF) levels of (A) CCL2, (B) CCL4, (C) CXCL10 and (D) CCL5 before (week 0) and after (weeks 4 and 16) immunization . Mean ± SD (Aged animals, *n* = 9; juveniles, *n* = 8).

CSF samples of aged and juvenile macaques were also used for multiplex analysis testing of various inflammatory cytokines and growth factors. Results for basic fibroblast growth factor (bFGF), granulocyte–macrophage colony-stimulating factor (GM-CSF), interferon-gamma (IFN-γ), interleukin (IL)-1b, IL-2, IL-4, IL-8, IL-10, IL-12 (p70), IL-15, IL-17a, CCL7 (monocyte chemotactic protein 3 (MCP-3), CXCL9 (monokine induced by gamma interferon; MIG), CCL3 (macrophage inflammatory protein 1a; MIP-1a), nerve growth factor (NGF), platelet-derived growth factor (PDGF)-bb, transforming growth factor beta (TGF-β), tumor necrosis factor alpha (TNF-α), and macrophage colony-stimulating factor (M-CSF) showed most measurements to be near or below the detection limit. Vascular endothelial growth factor (VEGF), plasminogen activator inhibitor 1 (PAI-1), IL-23, CXCL11 (IFN-inducible T-cell α chemoattractant; I-TAC), macrophage migration inhibitory factor (MIF), IL-6, and sCD40 were detectable, but exhibited no significant changes over time or between age groups. Analysis of CCL2 (MCP-1) revealed no significant difference at any time-point between juvenile and aged animals, but there was a significant decrease over time in the aged animals (Figure 8A). CCL4 (MIP-1b) concentrations were significantly higher in the aged cohort at the pre-immunization time-point, but this significant difference disappeared at the post-immunization follow-up time-points (Figure 8B). Analysis of CXCL10 (IFN-γ-induced protein 10 (IP-10) and CCL5 (regulated upon activation, normally T-cell expressed, and presumably secreted; RANTES) showed a significant effect of age in the repeated measures two-way ANOVA (CXCL10, *P* < 0.05; CCL5, *P* < 0.01) with overall higher levels in the aged animals but no significant trend over time (Figure 8C,D).

## Discussion

Active and passive anti-Aβ immunization strategies are among the most aggressively tested therapeutic approaches in the battle against AD. After disappointing early results in human clinical trials, several groups have advocated a transition to preventive immunization strategies, that is, vaccination at a preclinical stage [13,16,41]. In rodent models, preventive immunization strategies were effective in diminishing the accumulation of Aβ in the brain and reducing cognitive impairment [2,6,9]. Although aged NHPs with existing AD-like pathology have been successfully vaccinated against Aβ [32], few aged NHPs without AD pathology have been evaluated, showing no differences in hippocampal levels of soluble and insoluble Aβ or cytokines (TNF-α, IL-1α, IL-1β) between Aβ- and control-immunized animals [42].

We chose a NHP model for our studies because of the close genetic relationship to humans, identical APP amino acid sequence, and natural development of neuritic amyloid plaques and amyloid angiopathy with age. However, we also recognize the differences between humans with AD and NHP models [43], such as less severe tau pathology, different binding characteristics to PiB, and as our data demonstrate, significant differences in CSF biomarkers. Compared with non-demented aged humans [20,21], the aged macaques in our study had lower CSF Aβ and total tau levels and higher tau:Aβ_42_ ratios. Plaques are seen in most rhesus macaques older than 21–23 years [44,45] and cynomolgus macaques older than 18–20 years [46,47]; data on plaque occurrence in pigtailed macaques are lacking. The animals in our study were right within the age range at which plaques are first seen in these species, but their degree of pathology was milder than expected from previous reports, and unlikely to be associated with cognitive symptoms. Based on the pathologic findings, all our animals would be considered to be in a preclinical state and good candidates for a preventive immunization trial. However, it could be argued that the absence of significant differences in the CSF biomarkers tau and Aβ_42_ and the tau:Aβ_42_ ratio between aged and juvenile control animals at study onset does not accurately reflect the case in humans, for whom the use of biomarkers is advocated for identifying preclinical disease. On the other hand, recent studies have highlighted the potential utility of YKL40 in a panel of CSF biomarkers to distinguish cognitively normal people from patients with very mild or mild dementia [18]. We observed a significant difference in CSF YKL40 levels between aged and juvenile animals, closely resembling the age- and disease-associated increases seen in humans [18,22], suggesting that our aged cohort had entered a phase of neuropathologic decline. 

There were no differences in the degree of Aβ deposi-tion between immunized and non-immunized aged macaques; however, our follow-up period of six months was probably too short to witness any significant effects on cerebral Aβ accumulation, in particular considering the large variability in Aβ pathology in this age group, the small group size, and the heterogeneous genetic background of our animals. Despite these limitations, more detailed analysis revealed significant changes in the composition of oligomer species in immunized versus non-immunized aged animals, with increased ratios of lower oligomer species, predominantly dimers. Our findings suggest that even in the absence of significant amyloid build-up in the brain, anti-Aβ antibodies shift parenchymal oligomeric composition to smaller species, thereby possibly promoting Aβ clearance from the brain. Interestingly, we found a significant positive correlation between increasing CSF Aβ_42_ levels post-immunization and strength of the CSF humoral immune response, suggesting that antibodies facilitate Aβ mobilization. This effect may be explained by CSF antibodies either promoting an Aβ shift from the brain parenchyma to the CSF or, alternatively, slowing Aβ clearance from the CSF compartment. As a similar effect was seen in both juveniles and aged animals with varying brain amyloid loads, the increased CSF Aβ levels appeared independent of brain Aβ levels. These results, however, are in contrast to a previous study that noted a decrease in CSF Aβ levels after immunization in NHPs with more advanced amyloid pathology [32].

Soluble oligomers are increased in human AD brain tissue, and are more strongly associated than fibrillar Aβ with synaptotoxicity and cognitive impairment [33,48-52]. In particular, toxicity has been attributed to dimers, but also to trimers, pentamers, and dodecamers [33,50,53,54]. Limited data from the first human active immunization trial (AN-1792) demonstrated that immunization reduced oligomer levels in the frontal cortex [55]. Mass spectrometric analysis of brain extracts from three immunized patients showed a preponderance of dimeric Aβ oligomers [56]. It is unclear if the shift in oligomer composition observed after immunization in our study would have any transient or permanent biologic effects. Coadministration of anti-Aβ antibodies with oligomers or treatment with oligomer-specific antibodies prevented the synaptotoxic effects of oligomers *in vitro* and *in vivo*[50,57-60], thus, it may be speculated that antibodies induced by immunization would capture oligomers and prevent any synaptotoxic side-effects mediated by more toxic amyloid species.

Amyloid clearance after immunization is mediated by microglia-dependent and -independent mechanisms [1,61-65]. Neuropathologic examination of brains from deceased participants of the human AN1792 trial found evidence of Aβ phagocytosis by microglia [66,67]. To explore the largely unknown role of microglia in preventive immunization, we used both longitudinal monitoring via repeated [^11^C]PK11195 PET imaging and CSF analysis, and histopathologic and PCR analyses at time of necropsy. These studies did not reveal any significant differences in microglial density or activation between immune responders, non-responders, and non-immunized aged control animals, but a subset of chemokines (CCL2, CCL4, and CXCL10) had either increased baseline CSF levels in aged animals and/or a decline post-immunization in aged but not juvenile animals. Elevation of these inflammatory markers in AD or even MCI has been previously described [68-70], but their relationship to oligomers is unclear. Further studies are needed to determine if the change in oligomer composition in our animals is linked to the modest decline in inflammatory markers after immunization.

A wide spectrum of macrophage differentiation states exists, which are chiefly categorized into classical (M1) and alternative (M2) activation states, with distinct species-specific gene expression patterns and biologic functions [71-74]. However, there are conflicting data in the literature as to which activation state may be more beneficial for Aβ phagocytosis [75-79]. We identified simultaneous expression of various M1 and M2 markers in the aged brain post-mortem, but did not detect any significant immunization-related changes, suggesting that preventive vaccination has no major effect on the cerebral M1:M2 balance.

A well-known phenomenon with aging is diminished function of both the adaptive and innate arms of the immune system. This includes an increased threshold for immune response induction, which may be overcome by supplementing a vaccine with an appropriate immunostimulatory adjuvant [80]. We chose MPL, a derivative of the lipid moiety of lipopolysaccharide, which targets toll-like receptor 4 (TLR4) [81], and is already in clinical use in several vaccines [82]. TLRs are evolutionarily conserved pathogen receptors, but their function and expression is also affected by immunosenescence [83]. It has therefore been speculated that modulation of the innate immune system with TLR ligands may overcome these age-related functional changes and improve vaccine effectiveness in the elderly [84]. Nevertheless, immunosenescence was still evident in our study with vaccinations markedly less effective in the aged cohort. Neither of the adjuvants (aluminum phosphate for tetanus toxoid or MPL for the Aβ vaccine), was able to overcome this age-related decline in the immune response. Since the initiation of our study, many novel vaccine modifications have been developed. It remains to be seen if improved vaccine designs will lead to a higher rate of elderly immune responders.

In our study, the first dose of the Aβ vaccine was administered simultaneously with the tetanus control vaccine, thus we cannot exclude the possibility that coadministration affected the immune response to either vaccine. Although vaccine coadministration can induce both positive and negative effects on vaccine immunogenicity, most studies show little effect of tetanus/diphtheria vaccine on the immune response to other coadministered antigens [85-87]. The vaccine dose in our study of 100 μg Aβ per injection was slightly higher (about three times) than that used in the human AN1792 trial [10] when adjusted for the differences in body weight, but lower than in previous NHP studies [32,42], supporting the overall clinical and translational relevance of our findings.

## Conclusions

In this study, we found that preventive Aβ immunization is a safe therapeutic approach without serious side-effects in our aged and juvenile NHPs. Immunization did not induce increased inflammation, neither of the autoimmune/encephalitic type nor of the innate arm of the immune system. Although no effect on overall brain Aβ levels was seen, a shift in the composition of soluble oligomers towards smaller species was observed, leading us to speculate that this shift might facilitate the removal of toxic Aβ species from the brain. Even if these changes are small, they may have a significant influence over time on the balance between Aβ accumulation and clearance.

## Abbreviations

Aβ: Beta-amyloid; AD: Alzheimer’s disease; BSA: Bovine serum albumin; CERAD: Consortium to Establish a Registry for Alzheimer's Disease; CSF: Cerebrospinal fluid; DMSO: Dimethyl sulfoxide; EDTA: Ethylene diamene tetraacetic acid; FOV: Field of view; FWHM: Full width at half maximum; GAPDH: Glyceraldehyde 3-phosphate dehydrogenase; GFAP: Glial fibrillary acidic protein; Iba-1: Ionized calcium-binding adapter molecule 1; IDO: Indoleamine 2,3 dioxygenase; M1: Classical macrophage activation; M2: Alternative macrophage activation; MCI: Mild cognitive impairment; M-CSF: Macrophage colony-stimulating factor; MES: 2-(N-morpholino)ethanesulfonic acid; MPL: Monophosphoryl lipid A; MRC1: Mannose receptor 1 (CD206); NHP: Non-human primate; PBS: Phosphate-buffered saline; PET: Positron emission tomography; PiB: Pittsburgh Compound B; PTGS2: Prostaglandin-endoperoxide synthase 2 (cyclooxygenase 2); SDS: Sodium dodecyl sulfate; SUVR: Standardized uptake value ratio; SWMR: Subcortical white-matter ratio; TBS: Tris-buffered saline; TBS-T: Tris-buffered saline plus Tween; TOI: Time of injection; TLR: Toll-like receptor; TSPO: Translocator protein; VOI: Volume of interest.

## Competing interests

GE Healthcare holds a license agreement with the University of Pittsburgh based on the PiB-PET technology described in this manuscript. Dr Mathis is co-inventor of PiB and thus has a financial interest in this license agreement. GE Healthcare provided no grant support for this study, and had no role in the design or interpretation of results or preparation of this manuscript. All other authors have no conflicts of interest with this work. They had full access to all of the data in the study, and take responsibility for the integrity of the data and the accuracy of the data analysis.
